# Supplementary material for: Human-Specific Suppression of Hepatic Fatty Acid Catabolism by RNA-Binding Protein HuR
Source: Noncoding RNA. 2025 Sep 1;11(5):65. doi: 10.3390/ncrna11050065 (PMC12452626; doi:10.3390/ncrna11050065)
Supplement: Supplementary file 1 [file ncrna-11-00065-s001.zip › ncrna-3731413-supplementary.pdf]

Supplementary Figure S1

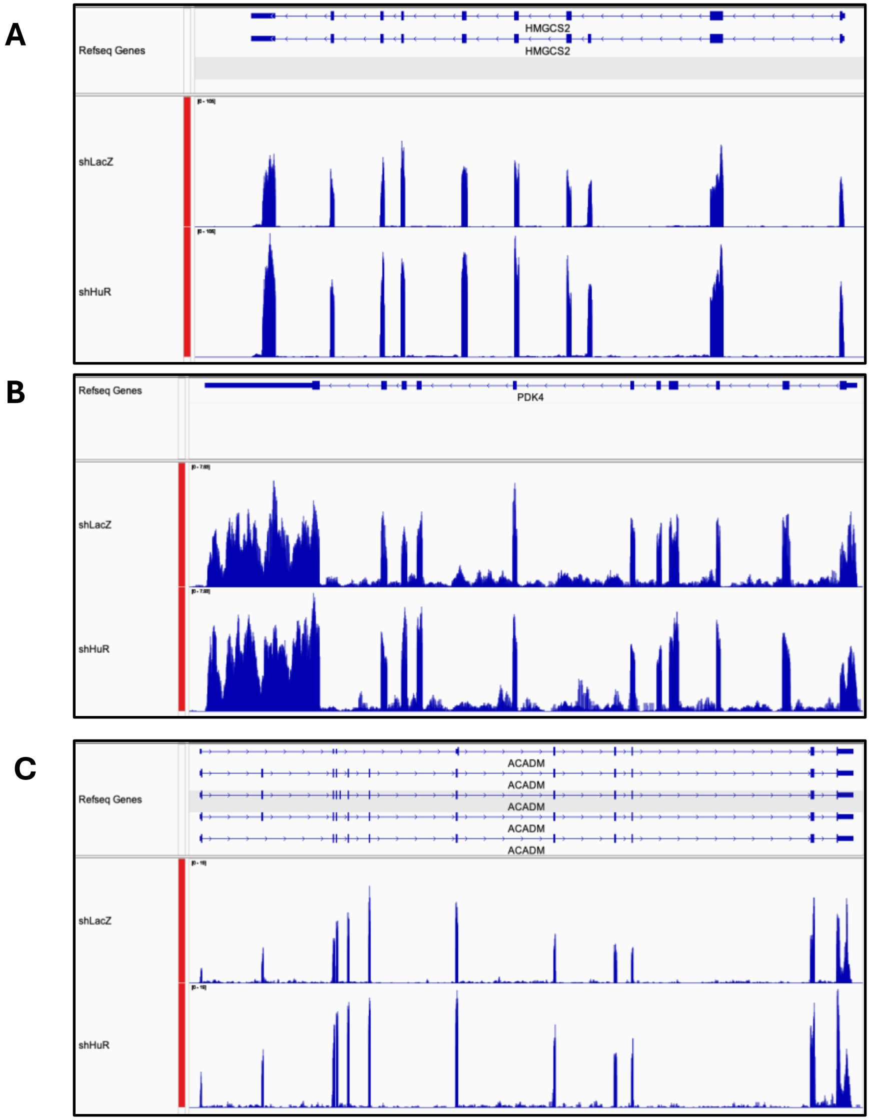

Supplementary Figure S1. A-C, RNA-seq alignment for human HMGCS2, PDK4, and ACADM using data from humanized livers with control (shLacZ) or human HuR knocking down (shHuR) as presented by IGV genome browser. CPM-normalized bigwig files were loaded to IGV genome browser, and the signal of 4 samples of each group were overlayed. The data range was adjusted in the same range by using "Group Autoscale" function.
